# The SIRT5-Mediated Upregulation of C/EBPβ Promotes White Adipose Tissue Browning by Enhancing UCP1 Signaling

**DOI:** 10.3390/ijms251910514

**Published:** 2024-09-29

**Authors:** Xiangyun Zhai, Liping Dang, Shiyu Wang, Chao Sun

**Affiliations:** College of Animal Science and Technology, Northwest A&F University, Yangling 712100, China; zhaixiangyun2016@126.com (X.Z.); dangliping2017@163.com (L.D.); 13607658576@163.com (S.W.)

**Keywords:** SIRT5, white adipose tissue browning, protein succinylation, fat synthesis, UCP1

## Abstract

Sirtuin 5 (SIRT5) plays an important role in the maintenance of lipid metabolism and in white adipose tissue browning. In this study, we established a mouse model for diet-induced obesity and the browning of white fat; combined with gene expression intervention, transcriptome sequencing, and cell molecular biology methods, the regulation and molecular mechanisms of SIRT5 on fat deposition and beige fat formation were studied. The results showed that the loss of SIRT5 in obese mice exacerbated white adipose tissue deposition and metabolic inflexibility. Furthermore, the deletion of SIRT5 in a white-fat-browning mouse increased the succinylation of uncoupling protein 1 (UCP1), resulting in a loss of the beiging capacity of the subcutaneous white adipose tissue and impaired cold tolerance. Mechanistically, the inhibition of SIRT5 results in impaired CCAAT/enhancer binding protein beta (C/EBPβ) expression in brown adipocytes, which in turn reduces the UCP1 transcriptional pathway. Thus, the transcription of UCP1 mediated by the SIRT5-C/EBPβ axis is critical in regulating energy balance and obesity-related metabolism.

## 1. Introduction

Adipose tissue is the central metabolic organ regulating energy balance. At present, it is generally believed that there are two types of adipose tissue, white adipose tissue (WAT) and brown adipose tissue (BAT), which differ significantly in morphology and function. WAT is used to store and release energy in mammals and is located mainly in the subcutaneous, omentum, tunica, and other parts. The intercellular loose connective BAT is rich in capillary and nerve fibers. The cells are smaller in size, with multiple smaller lipid droplets in the cytoplasm and more mitochondria. BAT is known for its ability to generate heat in cold environments, and its activity has been shown to be associated with weight management, glucose metabolism, and a lower risk of cardiovascular disease [1,2]. Notably, scattered brown-like adipocytes were found in WAT under the skin of mice after cold stimulation or adrenergic receptor treatment; these cells were named “beige fat” [3]. When unstimulated, beige fat is similar to white adipocytes in that it is a single-vesicular adipocyte with very low levels of intracellular uncoupling protein 1 (UCP1), the main function of which is to store energy. When the body is stimulated by the sympathetic nervous system, such as when experiencing cold stimulation or after exercise, the morphological and biological characteristics of beige fat cells change [4]. Many small fat droplets are scattered within the cells, the UCP1 levels and oxidative phosphorylation efficiency are increased, and its respiratory capacity is higher than that of typical brown fat. Its main role is in adaptive thermogenesis. Studies have shown that various drugs, metabolites, and signaling pathways regulate the beige process, which in turn affects the thermogenesis of adipose tissue and the homeostasis of whole-body energy metabolism [5,6,7]. Therefore, pharmacological studies aimed at stimulating BAT have good prospects in the treatment of metabolic diseases such as obesity and diabetes.

The brown adipocyte inner mitochondrial membrane contains UCP1, which breaks down oxidative respiration and ATP synthesis to produce heat energy expenditure, and it is considered a marker gene for BAT [8]. Over the years, several major regulators of brown fat have been identified, including transcriptional regulation, epigenetic regulation, non-coding RNA regulation, and metabolic rearrangements [9]. More than 30 transcription factors have been identified that affect the formation and function of brown and beige fat. Most of them are dependent on three core regulators, namely, peroxisome proliferator-activated receptor γ (PPARγ), PPARγ coactivator 1 α (PGC1α), and positive regulatory domain zinc finger protein 16 (PRDM16) [10,11,12,13]. For example, PGC-1 α is highly expressed in BAT compared with WAT, activating the cAMP-dependent thermogenesis of the adipocyte program; however, the deletion of PGC-1 α does not result in impaired fat browning. In contrast, the degradation of PRDM16 results in a loss of brown adipocyte identity. PPAR γ is indispensable for the function of WAT and BAT, and it induces the browning process of WAT mainly by cooperating with PRDM16. In addition to the three major transcription factors, the CCAAT enhancer-binding protein (C/EBP) is currently receiving attention as a transcription factor that positively regulates the browning of white fat [14,15]. The results showed that C/EBPβ and PRDM16 formed a transcription complex and induced the differentiation of brown adipocytes [9,14]. Thus, we can target the transcriptional activation of one or more core transcription factors for the directional regulation of beige fat formation.

Sirtuins (SIRTs) are NAD+-dependent protein deacetylases, which are highly expressed in BAT and WAT and regulate energy metabolism. The results show that SIRT3 and SIRT5 are closely related to adaptive thermogenesis. In particular, SIRT5 has been shown to be necessary for BAT formation in mice [16]. The specific knockout of SIRT5 in BAT leads to a sharp rise in protein succinylation and malonylation, which in turn affects the function of UCP1, triggering systemic energy metabolic disorders and impaired thermogenesis [17]. In contrast, treatment with the SIRT5 inhibitor MC3482 was found to stimulate brown adipose formation in 3T3L1 cells [18]. Another study in bovine preadipocytes showed that SIRT5 inhibited cellular adipogenic differentiation and triglyceride accumulation, increasing the proportion and diversity of unsaturated fats [19]. In summary, SIRT5 functions differently in different models and cells, which warrants further investigation. Although previous reports have demonstrated that SIRT5 promotes UCP1 expression, the molecular mechanism of its transcriptional activation of UCP1 is unclear. In the present study, we demonstrate that SIRT5 knockdown in mice exacerbates high-fat diet-induced obesity and lipid metabolic disorders; similarly, the knockdown of SIRT5 in β3-adrenergic receptor (β3-ar) agonist-activated white-fat-browning mice inhibits inguinal white adipose tissue beige transformation and adaptive thermogenesis, exacerbating the succinylation modification of proteins (there was no effect on acetylation). Mechanistically, SIRT5 interacts with C/EBPβ to activate the transcription of *Ucp1*, which in turn affects energy metabolism and thermogenesis.

## 2. Results

### 2.1. Sirt5 Deficiency Leads to Obesity and Metabolic Inflexibility in Mice

**Figure 1:**
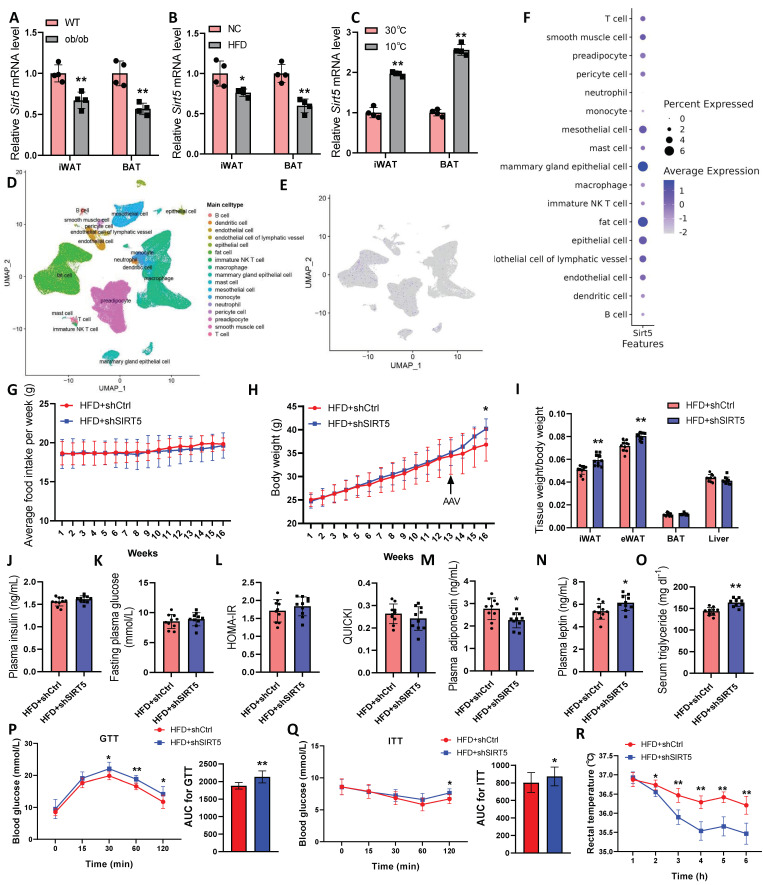
Effects of SIRT5 on body weight and glucose and lipid metabolism in HFD-induced obese mice. (**A**,**B**): mRNA levels of *Sirt5* in iWAT and BAT in *ob/ob* mice (**A**) or HFD-induced obese mice (**B**) (*n* = 4). (**C**): mRNA levels of *Sirt5* in iWAT and BAT of mice stored at 30 °C or 10 °C for 10 days (*n* = 4). (**D**–**F**): Analysis of *Sirt5* expression in GSE176171 dataset. (**G**,**H**): Average weekly food intake (**G**) and body weight (**H**) of each mouse (*n* = 10). (**I**): Tissue weight/body weight of mice in each group (*n* = 10). (**J**–**O**): Levels of insulin (**J**), glucose (**K**), HOMA-IR and QUICKI index (**L**), adiponectin (**M**), leptin (**N**), and triglyceride (**O**) in the blood of mice in each group (*n* = 10). (**P**,**Q**): GTT and ITT analysis of mice in each group (*n* = 10). (**R**): Rectal temperature of mice after acute exposure at different times (*n* = 10). HOMA-IR: homeostatic model assessment for insulin resistance; QUI-CKI: quantitative insulin sensitivity check index; AUC: area under the curve. * *p* < 0.05, ** *p* < 0.01, Student’s *t*-test (*n* ≥ 10), Mann–Whitney U test (*n* < 10).

To explore the potential physiological role of SIRT5, we performed a qPCR analysis of iWAT and BAT in obese mice. *Sirt5* is regulated by obesity in both BAT and iWAT, with a decreased expression found in genetically obese *ob/ob* mice (Figure 1A) and also in HFD-induced mice (Figure 1B). We kept the mice at 30 °C or 10 °C for 10 days and then tested the expression of *Sirt5*. In BAT and iWAT, 10 days of cold exposure significantly upregulated *Sirt5* expression (Figure 1C). Further, we analyzed the mouse single-nucleus RNA-seq data set in GSE176171 [20]. The results showed that *Sirt5* was highly expressed in mouse adipocytes and mammary epithelial cells (Figure 1D–F), suggesting that SIRT5 has a strong regulatory effect on fat. Next, we injected an shRNA adeno-associated virus targeting *Sirt5* into HFD-induced obese mice through an intraperitoneal injection. The results showed that the knockdown of *Sirt5* significantly promoted HFD-induced increases in body weight, iWAT, and eWAT in mice, with no difference in food intake between the two groups (Figure 1G–I). Further, *Sirt5*-knockdown mice showed marked abnormalities in glucose and lipid metabolism, manifested by increased serum triglyceride and leptin levels, glucose intolerance, and decreased insulin sensitivity and adiponectin levels (Figure 1J–Q). Interestingly, *Sirt5* knockdown mice exhibited a blunted response to cold stimulation, exhibiting a lower core temperature after 6 h at 4 °C (Figure 1R), suggesting that these mice have certain defects in activating adaptive thermogenesis. These data suggest that the loss of *Sirt5* exacerbates HFD-induced obesity and metabolic inflexibility.

### 2.2. Excessive Succinylation Due to Sirt5 Deficiency Leads to Increased Fat Synthesis in Mice

**Figure 2:**
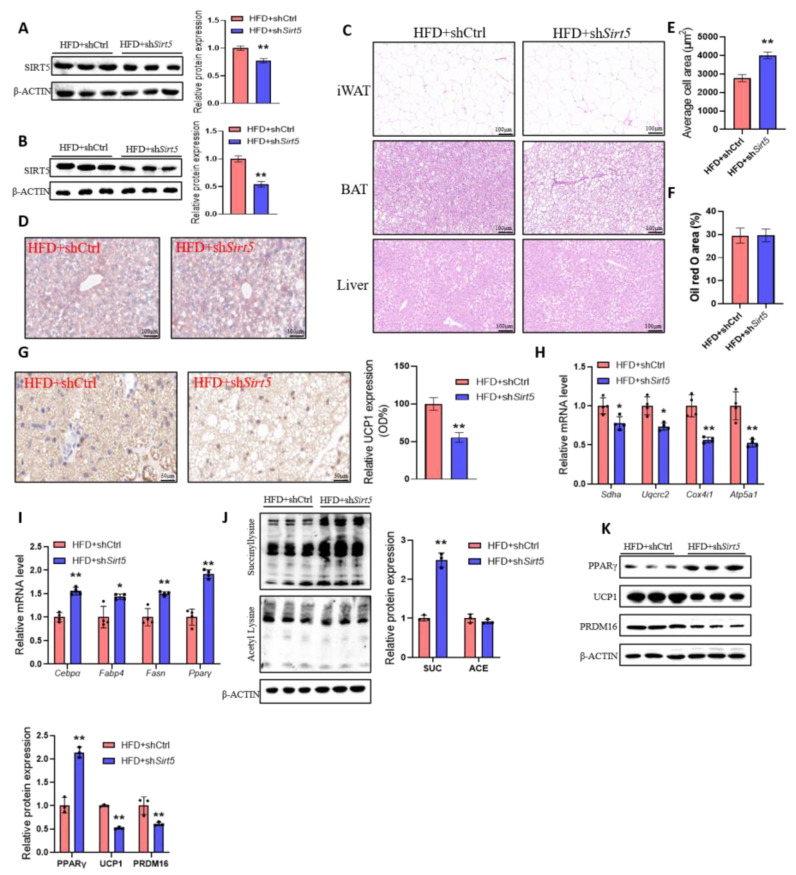
Effect of SIRT5 deletion on HFD-induced adipogenesis and browning of white fat in mice. (**A**,**B**): The protein levels of SIRT5 in BAT (**A**) and iWAT (**B**) of mice in each group (*n* = 3). (**C**): H and E staining of iWAT, BAT, and liver of mice in each group (*n* = 3). (**D**): The livers of mice in each group were stained with oil red O (*n* = 3). (**E**): Average cell area of subcutaneous iWAT in each group. (**F**): The area of liver stained with oil red O in each group. (**G**): Immunohistochemical staining of UCP1 in BAT of mice in each group (*n* = 3). (**H**): mRNA levels of mitochondrial-complex-related genes in mouse BAT (*n* = 4). (**I**): mRNA levels of lipid-synthesis-related genes in mouse iWAT (*n* = 4). (**J**,**K**): Western blot of iWAT from each group of mice (*n* = 3). * *p* < 0.05, ** *p* < 0.01, Mann–Whitney U test.

To test the effect of adeno-associated virus knockdown, we examined SIRT5 protein levels in mouse adipose tissue. The results showed that SIRT5 levels in both BAT (Figure 2A) and iWAT (Figure 2B) were significantly lower in the sh*Sirt5* group compared with the control group. The histological analysis of the adipose tissue revealed that *Sirt5*-knockdown iWAT contained larger adipocytes (Figure 2C,E); meanwhile, the BAT of sh*Sirt5* mice had larger lipid droplets compared with the BAT of the control group (Figure 2C). However, there was no significant difference in hepatic steatosis between the two groups (Figure 2D,F). Furthermore, we observed lower *Ucp1* expression in the BAT of *Sirt5* knockdown mice (Figure 2G). Consistent with this, the SIRT5 knockdown of BAT resulted in an impaired expression of genes associated with mitochondrial function (Figure 2H). In addition, *Sirt5* knockdown in iWAT significantly increased the expression of genes related to lipid synthesis (Figure 2I). We further found that the level of succinylation modification was higher in iWAT with *Sirt5* knockdown, but the level of acetylation did not change (Figure 2J); the protein expression levels of PRDM16 and UCP1 were also significantly reduced in the iWAT of *Sirt5*-inhibited mice (Figure 2K), with increased protein levels of PPARγ. These results suggest that *Sirt5* inhibits white-fat-browning progression by increasing the succinylation of adipose tissue and promoting the lipid synthesis of iWAT.

### 2.3. Excessive Succinylation Induced by Sirt5 Deletion Results in Impaired Browning of White Fat

**Figure 3:**
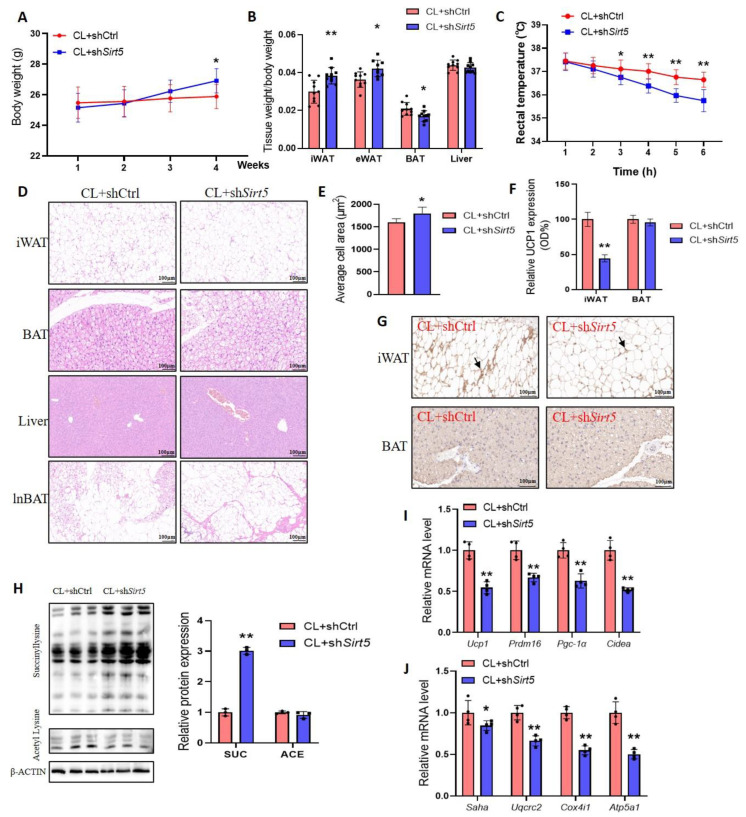
Effect of SIRT5 knockdown on browning of white fat in mice. (**A**): Body weight of *Sirt5* knockdown mice stimulated by CL (*n* = 10). (**B**): Tissue weight/body weight of mice in each group (*n* = 10). (**C**): Acute stimulation of rectal temperature in mice (*n* = 4). (**D**): H and E staining of iWAT, lnBAT, BAT, and liver of mice under the same treatment (*n* = 3). (**E**): Average cell area of subcutaneous iWAT in each group. (**F**,**G**): Immunohistochemical staining of UCP1 in iWAT and BAT of mice in each group (*n* = 3), arrows indicate detected UCP1. (**H**): Western blot of BAT from each group of mice (*n* = 3). (**I**,**J**): mRNA levels of mitochondrial-complex-related genes (**I**) and browning genes (**J**) in mouse BAT (*n* = 4). CL: CL316, 243; SUC: succinyllysine; ACE: acetylysine. * *p* < 0.05, ** *p* < 0.01, Mann–Whitney U test.

To further explore the metabolic regulation of UCP1 by SIRT5, we treated *Sirt5*-knockdown mice with a highly potent and selective β3-adrenergic receptor agonist (CL316243, CL). The results showed that the knockdown of *Sirt5* under CL treatment increased body weight and white adipose tissue weight in mice (Figure 3A,B). When sh*Sirt5* and control mice were subjected to acute cold exposure under ad libitum feeding conditions, mice in the sh*Sirt5* group exhibited poorer cold resistance compared with the control group, with the loss of body temperature in the two groups at 6 h varying by approximately 1.8 degrees (Figure 3C). Histological analyses of adipose tissue and the liver revealed that the *Sirt5* knockdown mice iWAT, lnBAT, and BAT contained larger adipocytes (Figure 3D,E). Further, we observed less multicolored beige fat and UCP1-positive staining area in the iWAT of mice in the sh*Sirt5* group (Figure 3F,G), but this phenomenon did not differ in BAT (Figure 3G). Interestingly, similar to HFD-induced mice, the levels of succinylation were significantly increased in *Sirt5*-knockdown mice under CL treatment, but the acetylation was unchanged (Figure 3H). Furthermore, the reduced transcription levels of mitochondrial-respiratory-chain-compound-related genes in *Sirt5* knockdown mice further led to a reduced expression of genes associated with the browning of white adipose tissue in mice (Figure 3I,J). The above results suggest that the deletion of *Sirt5* leads to the increased succinylation of BAT, which in turn impairs the CL-induced browning of white fat and the regulation of lipid metabolism.

### 2.4. SIRT5 Ameliorates Obesity-Related Metabolic Dysfunction in Mice by Regulating C/EBPβ Transcription

**Figure 4:**
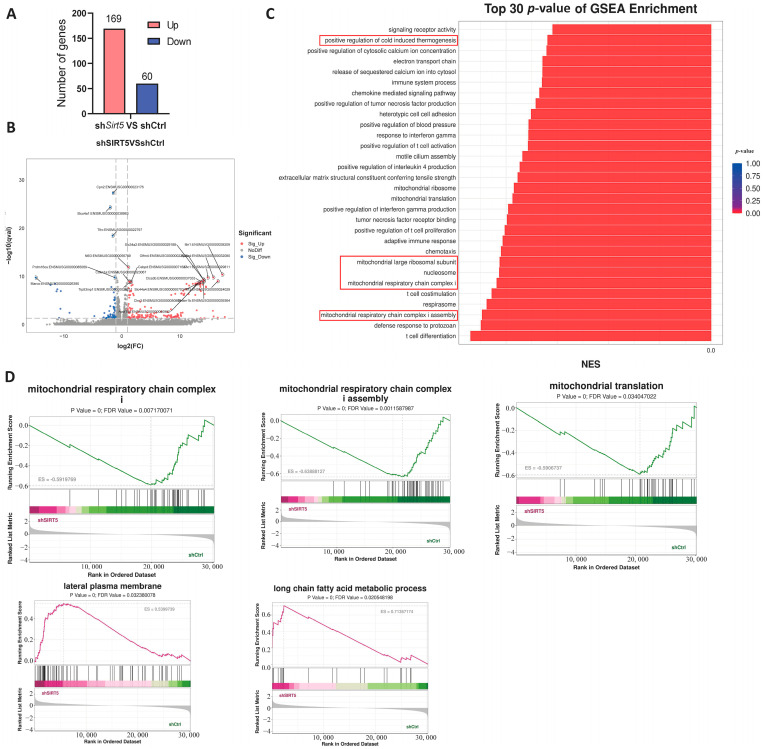
RNA-seq analysis of BAT in *Sirt5* knockdown mice. (**A**): Different genes were significantly upregulated or downregulated in each group (*n* = 4). (**B**): Volcano map of differentially expressed genes. (**C**): Functional enrichment analysis of differentially expressed genes. (**D**): GSEA analysis of mice in each group.

**Figure 5:**
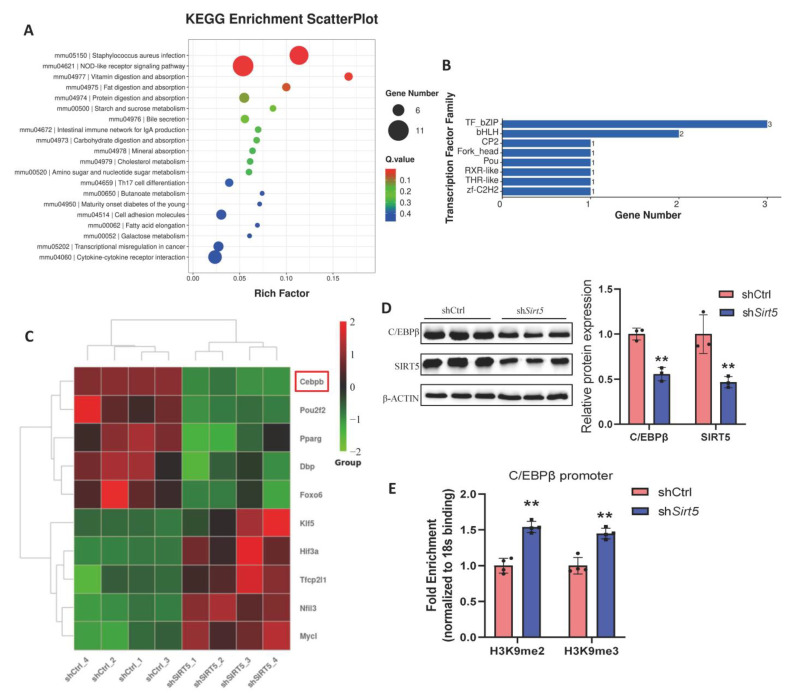
Effect of *Sirt5* knockdown on transcription factors in BAT of mice. (**A**): KEGG analysis of mice in each group. (**B**): The number and classification of different transcription factors in each group. (**C**): Heat map of differential transcription factors. (**D**): Immunoblot analysis of brown adipocytes in different treatments (*n* = 3). (**E**): Binding levels of h3k9me2 and h3k9me3 in the C/EBPβ promoter region (*n* = 4). ** *p* < 0.01, Mann–Whitney U test.

To further investigate the mechanism of action of *Sirt5* deletion in mice leading to obesity and impaired thermogenesis, we performed the RNA sequencing of BAT from SIRT5-knockdown and control mice. We identified 229 differentially expressed genes from the sh*Sirt5* group compared with the shCtrl group, including 169 upregulated genes and 60 downregulated genes (Figure 4A). Among the top-ranked genes were thermogenesis-related genes (PRDM16) and inflammation-related genes (Marco) (Figure 4B). A functional enrichment analysis of 229 differentially expressed genes due to *Sirt5* knockdown revealed the enrichment of several mitochondrial pathways, including complex I biosynthesis, and the positive regulation of cold-induced thermogenesis, mitochondrial ribosome, and translation (Figure 4C red square, D). The GSEA analysis showed that the long-chain fatty acid metabolic process and lateral plasma membrane were significantly upregulated in the sh*Sirt5* group compared with the control group (Figure 4D). The KEGG analysis of these candidate genes suggested that lipid metabolism (including fatty acid elongation and fat digestion and absorption) and inflammatory pathways are the signaling pathways that undergo the most significant changes (Figure 5A). To screen for *Sirt5*-activated transcription factors, we analyzed transcription factor expression profiles in these candidate genes. Histograms show the family distribution of differentially expressed transcription factors (Figure 5B). Among these transcription factors, we observed that C/EBPβ was the first downregulated transcription factor in the sh*Sirt5* group compared with the control group (Figure 5C). To validate these candidates, we examined the expression of C/EBPβ in *Sirt5*-knockdown C3H10T1/2 cells. As expected, SIRT5 significantly downregulated the protein level of C/EBPβ (Figure 5D). Other studies have shown that SIRT5 is involved in brown adipogenic gene activation by increasing the levels of the histone markers H3K9me2 and H3K9me3 [21]. We therefore performed CHIP assays in SIRT5-knockdown cells. The CHIP results showed that sh*Sirt5* group cells had significantly increased binding levels of h3k9me2 and h3k9me3 at the promoter region of C/EBPβ compared with those of the control group (Figure 5E). The above results indicate that the knockdown of *Sirt5* leads to the enrichment of H3K9me2 and H3K9me3 at the promoter region of C/EBPβ, which inhibits the expression of C/EBPβ, resulting in impaired mitochondrial function and the browning of white fat.

### 2.5. Sirt5 Represses the Transcription of UCP1 via C/EBPβ

**Figure 6:**
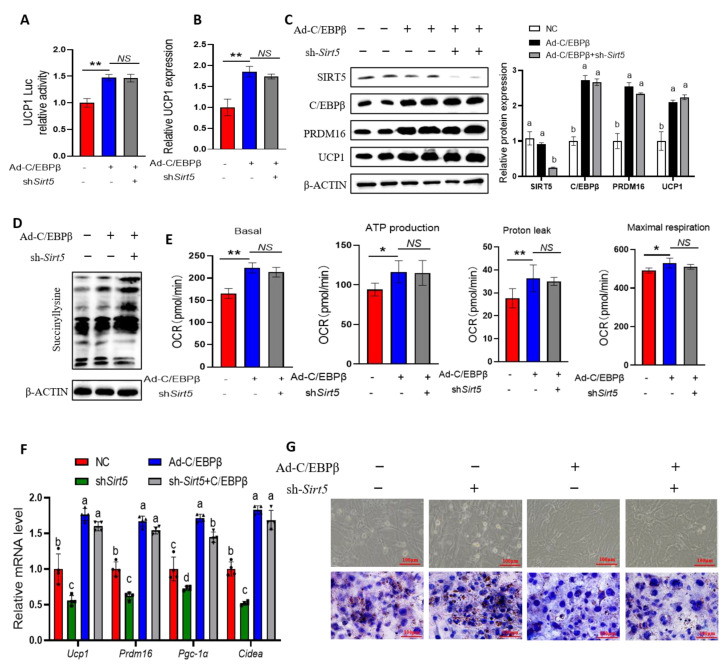
The activation of UCP1 transcription by SIRT5 is dependent on C/EBPβ. (**A**): Reporter analysis of C3H10T12 cells after *Sirt5* knockdown and transfection with C/EBPβ vector (*n* = 3). (**B**): UCP1 expression in C3H10T12 cells transfected with C/EBPβ vector after SIRT5 knockdown (*n* = 3). (**C**,**D**): Immunoblot analysis of C3H10T12 cells treated with the same treatment. (**E**): Continuous measurement of oxygen consumption in C3H10T12 cells treated with the same treatment (*n* = 3). (**F**): The mRNA levels of thermogenic genes in C3H10T12 cells treated with the same treatment (*n* = 4). (**G**): Oil red O staining was performed on C3H10T12 cells under the same treatment. OCR: O_2_ consumption rate. * *p* < 0.05; ** *p* < 0.01; *NS*: not significantly different. Lowercase letters indicate statistical significance, and groups with different letters indicate *p* < 0.05.

As C/EBPβ has been shown to promote UCP1 gene expression [22,23,24], we analyzed whether the effect of SIRT5 on UCP1 transcriptional activity was dependent on C/EBPβ. We analyzed the transcriptional effect of C/EBPβ translocation on the *Ucp1* gene under SIRT5 inhibition using a dual-luciferase reporter system. As shown in Figure 6A, the luciferase reporter assays showed that C/EBPβ overexpression promoted *Ucp1* transcription, whereas the knockdown of *Sirt5* did not reduce UCP1 transcription. The expression of *Ucp1* was consistent with the dual-luciferase report (Figure 6B). Further, we observed that the SIRT5 promotion of the protein expression of UCP1 and PRDM16 was similarly dependent on C/EBPβ (Figure 6C), whereas this effect was associated with the direct regulation of succinylation by SIRT5 (Figure 6D). To further confirm the role of the SIRT5-C/EBPβ axis in regulating adipose thermogenesis in vitro, we measured respiratory effects with a hippocampal extracellular flux analyzer. We measured basal OCR, ATP production, proton leakage, and maximal respiratory volume, showing that SIRT5 promotes oxygen consumption in brown adipocytes through C/EBPβ action (Figure 6E). The change in the mRNA level of browning-related genes was also consistent with the trend of oxygen consumption (Figure 6F). We next confirmed the browning effect of the SIRT5-C/EBPβ axis in C3H10T1/2 cells. Oil red O staining and visual estimation by phase contrast microscopy showed that *Sirt5*-inhibited adipocytes contained more and larger lipid droplets than the control cells, but this effect disappeared after C/EBPβ overexpression (Figure 6G). Thus, the above results demonstrate that SIRT5-C/EBPβ signaling regulates UCP1 expression by controlling the transcription of C/EBPβ, thereby affecting adipocyte thermogenesis.

## 3. Discussion

Our results show that the knockdown of *Sirt5* exacerbates HFD-induced obesity and lipid metabolic disorders characterized by increased adipose tissue deposition, insulin resistance, and impaired adaptive thermogenesis. Specifically, the reduced expression of *Ucp1* and the impaired mitochondrial respiratory chain confirmed that *Sirt5* knockdown was able to inhibit white fat browning; this effect was associated with increased protein succinylation modification. SIRT5 is located in the mitochondria and is essential for cellular energy metabolism. Shuai et al. demonstrated that SIRT5 is required for BAT formation, mainly because SIRT5 reduces intracellular α-ketoglutaric acid concentrations, resulting in elevated H3K9me2 and H3K9me3 levels in the promoter regions of the PPAR γ and PRDM16 loci [16]. Furthermore, studies in obese model mice have shown that the overexpression of *Sirt5* ameliorates metabolic abnormalities in *ob/ob* mice, whereas malonylation and succinylation are potential targets for therapy [25]. Mutations in succinylation and malonylation sites of UCP1 significantly reduce the stability and activity of the protein. The deletion of *Sirt5* leads directly to a sharp increase in UCP1 succinylation, which in turn reduces mitochondrial energy metabolism and thermogenesis [17]. These data are consistent with our findings. In addition to thermogenesis genes, our results suggest that SIRT5 regulates the expression of genes related to adipogenesis, including FASN, C/EBP α, and FABP4. Similar studies have found that SIRT5 downregulates the expression of lipogenic differentiation genes by inhibiting the mitogen-activated protein kinase pathway, resulting in reduced lipid deposition [26]. Thus, we propose that SIRT5 plays a role in regulating lipid metabolism and brown adipocyte differentiation by affecting the expression of thermogenic genes, but some potential mechanisms of its role warrant further investigation.

In the past few decades, most studies have demonstrated that the β3-adrenergic receptor, which is present in adipose tissue, is a typical pathway for the activation of thermogenic fat [27]. This is because under sympathetic nervous system control, UCP1 acts as an uncoupled oxidative phosphorylation that increases ATP production in the mitochondria, mainly through the mitochondrial mediation of β-adrenalin [28,29]. Thus, β3-adrenergic receptor selective agonists such as CL are often used as stimulators to stimulate fat thermogenesis and lipid mobilization. In our study, we found that the deletion of SIRT5 in mice under CL stimulation resulted in the impaired beige staining of white fat and decreased thermogenesis. This effect is mainly related to decreased mitochondrial function and energy metabolism. Further analysis of SIRT5-mediated transcription factors revealed that both C/EBPβ and PPAR γ were regulated by SIRT5. Previous studies have revealed that SIRT5 activates its expression by promoting the methylation of the PPAR γ promoter region [10,16,30]. However, the regulatory relationship between SIRT5 and C/EBPβ has not been studied, and this is worth further exploration.

The molecular basis of UCP1 transcriptional regulation has been thoroughly studied. The proximal region of the transcription start site of UCP1 includes the CREB (cAMP response element binding protein) binding site and a C/EBP binding site capable of responding to both C/EBP α and C/EBPβ [31]. In addition, there is a strong enhancer region approximately 2.5 kb upstream of the transcriptional start site in mice, which includes receptor-binding elements for PPAR α and PPARγ [32,33]. These regulatory structures confer specific expressions of UCP1 in BAT and directly determine the differentiation and thermogenesis of brown adipocytes. Notably, our results found a positive correlation between SIRT5 and the molecular level of C/EBPβ, and the knockdown of SIRT5 led directly to the inhibition of methylation in the C/EBPβ transcriptional promoter region, which in turn affected the expression of UCP1. This effect disappeared after the overexpression of C/EBPβ, indicating that the regulatory effect of SITR5 on UCP1 was dependent on C/EBPβ. Other studies have shown that C/EBPβ acts as a transcriptional activator in the core promoter region of SIRT5 [34]. Our study further links the mechanism by which SIRT5 regulates UCP1 transcription to C/EBPβ, illustrating that in addition to PPARγ, SIRT5 can regulate UCP1 by mediating C/EBPβ, which in turn affects white fat browning and energy metabolism.

## 4. Materials and Methods

### 4.1. Mouse Experiment

All animal experiments were approved by and performed in accordance with the guidelines and regulations of the Animal Ethics Committee (Northwest A&F University, Yangling, Shaanxi, China). Seven-week-old male mice with a C57BL/6 background were purchased from the Laboratory Animal Center of the Fourth Military Medical University (Xi’an, China) and housed on a 12/12 h light/dark cycle with free access to food and water. Leptin knockout (*ob/ob*) mice were previously stored in our laboratory [35]. To construct a diet-induced obesity model, mice were fed a high-fat diet (HFD; protein, 20%; fat, 60%; carbohydrate, 20%) purchased from Xietong Pharmaceutical Bio-engineering Co., Ltd. (Nanjing, China). Adeno-associated virus (AAV) *Sirt5* shRNA plasmid (GV390) was purchased from GenechemCo., Ltd. (Shanghai, China) for a knockdown assay. The targeting sequence was GCACTGTTGCCGAGAACTATA. Twelve weeks after HFD induction, all mice were randomly divided into two groups of ten mice each. The constructed sh*Sirt5* or control vector AAV was administered to mice (1 × 10^8^ PFU) via intraperitoneal injection once a week for four weeks. To construct a white-fat-browning model, each mouse was intraperitoneally injected with a β3-adrenergic receptor agonist (CL316243, 1 mg/kg/d) once daily for seven consecutive days. During the experiment, body weight was monitored weekly, and food intake was measured for each mouse every two days. After anesthesia, the mice were sacrificed by cervical dislocation. Epididymal WAT (eWAT), inguinal WAT (iWAT), BAT, and the liver were removed, and the following steps were undertaken: (1) the testes were first found by cutting through the lower abdomen of the mice, and then the eWAT was stripped and cut down along the vas deferens. (2) Skin and muscle were passively separated with ophthalmic scissors, and then the skin was cut lengthwise to expose the iWAT. The inguinal lymph nodes were stripped, and the tissue was cut out. (3) The skin on the back of the mice was sheared to expose the symmetrical BAT in the scapular region. The BAT was further sheared and the white adipose tissue covering the surface was removed. All tissues were removed and rinsed in ice-cold saline to remove hair and blood, which were frozen in liquid nitrogen or fixed in 4% paraformaldehyde (Servicebio, Wuhan, China).

### 4.2. Cell Culture

C3H10T1/2 cells are derived from mesenchymal stem cells of mice embryos and have the ability to differentiate into brown adipocytes [16,36]. C3H10T1/2 cells were purchased from Procell Life Science & Technology Co. Ltd. (Wuhan, China). C3H10T1/2 cells were maintained in a DMEM medium (Gibco, San Diego, CA, USA) containing 10% fetal bovine serum (FBS, Sigma-Aldrich, St. Louis, MO, USA) and 1% penicillin-streptomycin (Gibco, San Dieago, CA, USA). Sh*Sirt5* and shCtrl AAV were transfected into C3H10T1/2 cells, which then induced cell differentiation. The CDS sequence of mouse C/EBPβ was cloned into a pLV-GFP-C1 virus expression plasmid for the detection of overexpression. To induce browning of the C3H10T12 cells, cells were differentiated in DMEM containing 10% FBS, 0.5 mM 3-isobutyl-1-methylxanthine, 1 μM dexamethasone (Solarbio, Beijing, China), and 10 μg/mL insulin (MCE, Shanghai, China) for two days. Cells were then further differentiated in DMEM containing 10% FBS and 10 μg/mL insulin until lipid droplets appeared. The cell culture medium was changed every two days.

### 4.3. Temperature Measurements

To induce cold stress, mice were transferred from a 25 °C environment to a 4 °C environment for 6 h with free access to water and food. The body temperature of the mice was measured using a rectal probe (TH-5 thermalert monitoring thermometer, Hedebio, Beijing, China) every hour.

### 4.4. Glucose and Insulin Tolerance Test

A glucose (Solarbio, Beijing China) tolerance test (GTT) and an insulin tolerance test (ITT) were performed according to the methods previously described by our laboratory [37]. Briefly, for GTT, mice were fasted overnight and injected intraperitoneally with 1.5 g glucose/kg body weight. For ITT, mice were given 1 U insulin/kg of body weight intraperitoneally after a 6-h fast. Blood glucose was then measured in each mouse at 0, 15, 30, 60, and 120 min, respectively. The area under the curve (AUC) was calculated by adding together the trapezoidal area of two adjacent time points.

### 4.5. Histological and Immunohistochemical (IHC) Analyses

The livers, iWAT, lymph node beige adipose tissue (lnBAT), and BAT of mice were fixed with 4% paraformaldehyde and then embedded in paraffin (Servicebio, Wuhan, China), and 5 μm thick sections were prepared. The sections were then stained with hematoxylin and eosin (H & E, Servicebio, Wuhan, China). In order to observe the steatosis of the liver, frozen liver slices were stained with oil red O (Solarbio, Beijing, China). The wax blocks of BAT and iWAT were cut to 5 μm thickness, and the samples were dewaxed and antigen-repaired successively. Sections were then placed in a blocking solution (Beyotime, Shanghai, China) and incubated with the corresponding primary antibody at 25 °C (anti-UCP1, dilution 1:200, AB209483, ABCAM, Cambridge, UK). Sections were rinsed with phosphate-buffered saline (PBS) and then incubated with the corresponding secondary antibody (Abmart, Shanghai, China) for 2 h. Histological data were collected using ImageJ software (v1.54). Positive area was quantified by staining area/total area.

### 4.6. Respiration Assays

The treated differentiated C3H10T12 cells were washed once with PBS and maintained in XF basal medium (Agilent, Beijing, China) containing 25 mM glucose (Agilent, Beijing, China), 1 mM pyruvate (Agilent, Beijing, China), and 2 mM L-l-glutamine (Agilent, Beijing, China) for 1 h. The oxygen consumption rate (OCR) was measured by adding together 5 μM of oligomycin (MCE, Shanghai, China), 5 μM of FCCP (MCE, Shanghai, China), and 5 μM of antimycin (MCE, Shanghai, China). The OCR was calculated automatically using hippocampal XF24 software (v2.4.2).

### 4.7. Dual-Luciferase Reporter Assay

The *Ucp1* promoter region of 4148 bp in mice was amplified by PCR and fused with a pGL-3 basic vector (Promega, Madison, WI, USA). Confluent C3H10T12 cells were infected with Ad-CREB/β or sh*Sirt5* adenovirus. Then, the cells were co-transfected with pGL3-*Ucp1* and pRL-TK using Lipo8000 (Beyotime, Shanghai, China). Luciferase activity was measured in the cell lysate using a dual-luciferase reporting system according to the manufacturer’s instructions (Beyotime, Shanghai, China).

### 4.8. Chromatin Immunoprecipitation (ChIP) Assay

*Sirt5*-knockdown-differentiated cells were collected for methylated ChIP assays. The assays were performed using an AB500 ChIP kit (ABCAM, Cambridge, UK) according to the manufacturer’s instructions. All enrichment changes were normalized to input, and 18s rRNA was used as a nonspecific binding site.

### 4.9. Real-Time Quantitative (qPCR) Analysis

Total RNA was extracted from tissues and cells using a Trizol reagent (Takara, Tokyo, Japan), and its concentration and purity were measured. cDNA was generated by the reverse transcription of a PrimeScript RT kit (Takara, Beijing, China). qPCR was performed in triplicate wells using SYBR Premix Ex Taqs (Takara, Beijing, China). β-actin was used as an endogenous control gene. The gene expression was analyzed using the 2^−ΔΔCT^ method. All specific primers used in this study are listed in Table 1.

### 4.10. Western Blotting

A protein lysis buffer (100 mM Tris-HCl (Solarbio, Beijing, China), pH 6.8, 100 mM DTT (Solarbio, Beijing, China), 1% SDS (Solarbio, Beijing, China), 10% glycerol (Solarbio, Beijing, China)) was used to extract proteins from cells and adipose tissue. The protein was separated using 10–12% sodium lauryl sulfate polyacrylamide gel electrophoresis (SDS-PAGE) (epizyme, Shanghai, China) and transferred to a PVDF membrane (Millibore, Burlington, MA, USA). PVDF membranes were blocked in PBS containing 5% skim milk for 2 h and then incubated with primary antibodies at 4 °C overnight. Primary antibodies were used as indicated: anti-SIRT5, 1:5000 dilution (GeneTex, San-Antonio, TX, USA); anti-UCP1, 1:200 dilution (Abcam, Cambridge, UK); anti-PPARγ, 1:5000 dilution, (Abcam, Cambridge, UK); anti-PRDM16, 1:5000 dilution, (Abcam, Cambridge, UK); anti-C/EBPβ, 1:5000 dilution (Santa Cruz, Dallas, TX, USA); anti-succinyllysine, 1:2000 dilution (PTM-BIOLAB, Hangzhou, China); anti-acetyl-lysine, 1:2000 dilution, (PTM-BIOLAB, Hangzhou, China). Membranes were washed with and incubated with secondary antibodies (Abmart, Shanghai, China) for 2 h. Immunoreactive proteins were subsequently visualized using the enhanced chemiluminescence (ECL, NCM, Suzhou, China) detection system (Millipore). Immunoblot quantification was performed using Image J (v1.54).

### 4.11. RNA Sequencing (RNA-seq) and Data Processing

Total RNA was extracted from shCtrl and shSIRT5 mouse BAT using TRIzol (Thermofisher, 15596018). The RNA quality was assessed using NanoDrop ND-1000 (NanoDrop, Wilmington, NC, USA), and cDNA libraries were constructed. It was double-end sequenced using Illumina NovaseqTM 6000 (LC BIO Technology Co., Ltd., Hangzhou, China) in a sequencing mode of PE1500 following standard operations. The FPKM values (Fragments Per Kilobase Million, normalized to the original read count of the gene) were used as the measure of gene expression, and the expression of genes in different samples was counted. The differentially expressed genes (fold change > 1.5, *p*< 0.05) were represented by a volcano plot. A cluster heatmap was used to display the differential gene expression patterns, and the differentially expressed genes were enriched by GO and KEGG. Based on the principle of GSEA analysis, GO items were enriched and analyzed. Significant pathway enrichment was set at *p* < 0.05. Based on the ANIMALTFDB database https://guolab.wchscu.cn/AnimalTFDB4/#/ (accessed on 17 January 2023), the genes in the gene expression profile were annotated with transcription factors, and the family distribution of differential transcription factors was analyzed.

### 4.12. Statistical Analysis

All experimental data were analyzed with the SPSS (v25.0) statistical software program. The data were analyzed for normality using the Shapiro–Wilk test. Data were described as mean ± SD and statistically analyzed using a two-tailed Student’s *t*-test (*n* ≥ 10) and the Mann–Whitney U test (*n* < 10) for shCtrl and sh*Sirt5* groups. If more than two groups of data were compared, a one-way analysis of variance (ANOVA) with multiple comparisons was applied. *p* < 0.05 was considered significant.

## 5. Conclusions

In conclusion, our work provides new theoretical data for the effect of the regulation of SIRT5 on brown adipocyte differentiation in vitro and the browning process in vivo. Our current data support an important role for SIRT5 in mitigating diet-induced obesity and promoting adaptive thermogenesis. Mechanistically, SIRT5 interacts directly with C/EBPβ, leading to the increased transcription of *Ucp1*, which in turn regulates beige and mitochondrial energy metabolism in adipocytes. These findings have important implications for SIRT5 as an effective target to improve metabolic processes in the treatment of obesity and other metabolic diseases.

## Figures and Tables

**Table 1:** The qPCR primers used in this study.

Gene Name	Primer
*Sirt5*	CTCCGGGCCGATTCATTTCC
	GCGTTCGCAAAACACTTCCG
*Sdha*	GGAACACTCCAAAAACAGACCT
	CCACCACTGGGTATTGAGTAGAA
*Uqcrc2*	AAAGTTGCCCCGAAGGTTAAA
	GAGCATAGTTTTCCAGAGAAGCA
*Cox4i1*	ATTGGCAAGAGAGCCATTTCTAC
	CACGCCGATCAGCGTAAGT
*Atp5a1*	TCTCCATGCCTCTAACACTCG
	CCAGGTCAACAGACGTGTCAG
*C/ebpβ*	TCGGGACTTGATGCAATCC
	AAACATCAACAACCCCGC
*Fabp4*	ACACCGAGATTTCCTTCAAACTG
	CCATCTAGGGTTATGATGCTCTTCA
*Fasn*	GGAGGTGGTGATAGCCGGTAT
	TGGGTAATCCATAGAGCCCAG
*Pparγ*	TTCCGAAGAACCATCCGATTG
	TTCCGAAGAACCATCCGATTG
*Ucp1*	ACTGCCACACCTCCAGTCATT
	CTTTGCCTCACTCAGGATTGG
*Prdm16*	CAGCACGGTGAAGCCATTC
	GCGTGCATCCGCTTGTG
*Pgc1-α*	TATGGAGTGACATAGAGTGTGCT
	CCACTTCAATCCACCCAGAAAG
*Cidea*	TGCTCTTCTGTATCGCCCAGT
	GCCGTGTTAAGGAATCTGCTG
*Beta-actin*	GGCTGTATTCCCCTCCATCG
	CCAGTTGGTAACAATGCCATGT

## Data Availability

The raw data supporting the conclusions of this article will be made available by the authors on request.
